# Towards in vivo estimation of reaction kinetics using high-throughput metabolomics data: a maximum likelihood approach

**DOI:** 10.1186/s12918-015-0214-7

**Published:** 2015-10-05

**Authors:** Weiruo Zhang, Ritesh Kolte, David L Dill

**Affiliations:** Department of Electrical Engineering, Stanford University, 450 Serra Mall, Stanford, CA94305 USA; Department of Computer Science, Stanford University, 353 Serra Mall, Stanford, CA94305 USA

**Keywords:** Relative error, Enzymatic reaction, Parameter estimation, Maximum likelihood, Error-in-all-measurements, *In vivo* data

## Abstract

**Background:**

High-throughput assays such as mass spectrometry have opened up the possibility for large-scale *in vivo* measurements of the metabolome. This data could potentially be used to estimate kinetic parameters for many metabolic reactions. However, high-throughput *in vivo* measurements have special properties that are not taken into account in existing methods for estimating kinetic parameters, including significant *relative* errors in measurements of metabolite concentrations and reaction rates, and reactions with multiple substrates and products, which are sometimes reversible. A new method is needed to estimate kinetic parameters taking into account these factors.

**Results:**

A new method, InVEst (*In Vivo* Estimation), is described for estimating reaction kinetic parameters, which addresses the specific challenges of *in vivo* data. InVEst uses maximum likelihood estimation based on a model where all measurements have relative errors. Simulations show that InVEst produces accurate estimates for a reversible enzymatic reaction with multiple reactants and products, that estimated parameters can be used to predict the effects of genetic variants, and that InVEst is more accurate than general least squares and graphic methods on data with relative errors. InVEst uses the bootstrap method to evaluate the accuracy of its estimates.

**Conclusions:**

InVEst addresses several challenges of *in vivo* data, which are not taken into account by existing methods. When data have relative errors, InVEst produces more accurate and robust estimates. InVEst also provides useful information about estimation accuracy using bootstrapping. It has potential applications of quantifying the effects of genetic variants, inference of the target of a mutation or drug treatment and improving flux estimation.

**Electronic supplementary material:**

The online version of this article (doi:10.1186/s12918-015-0214-7) contains supplementary material, which is available to authorized users.

## Background

High-throughput assays such as mass spectrometry are improving rapidly, which creates an opportunity for large scale *in vivo* measurements of the metabolome. Those *in vivo* data could enable estimation of kinetic parameters of metabolic reactions which are hard to estimate using *in vitro* data.

Metabolic reactions are normally enzyme-catalyzed reactions, and quantitative estimates of their kinetic parameters could be very useful. Knowledge of kinetic parameters allows estimation of reaction rates directly from concentration measurements. Comparing the estimated kinetic parameters of a reaction in the wild type and mutant cells permits quantification of the effects of genetic variants, which may change the abundance or activity of a metabolic enzyme. Similarly, the effect of a drug that targets a particular enzyme could be estimated. If parameters can be estimated for many reactions in a pathway, it would enable inference of the target of a mutation or drug treatment – if the estimates show that one enzyme is particularly strongly affected, that enzyme is probably the target. Finally, estimated parameters also allow estimation of maximum reaction rates, which can then be used as constraints to improve flux balance analysis [1].

We explore the central problem of how to estimate the kinetic parameters of individual reactions using *in vivo* high-throughput measurements of metabolite concentrations and reaction rates at steady state, obtained by mass spectrometry or by nuclear magnetic resonance. The method requires metabolite concentration and reaction rate data in multiple experiments under varying conditions. For example, data could consist of several experiments obtained by perturbing the system through changes in nutrient media, drug treatment, or genetic alterations. From such data, the kinetics of many individual reactions can potentially be estimated.

Enzyme kinetic parameters have been measured for at least a century [2]. The basic method involves mixing a measured amount of substrate and enzyme, and measuring the concentration of product at various points in time, creating a *progress curve* [3]. In this setting, the experimenter has control over the initial concentrations of enzyme and substrate and thus can obtain relatively accurate measurements for concentrations. Although the experimental conditions are not at steady state, the mathematical formula for the kinetics can be simplified to the familiar Michaelis Menten kinetics by assuming that some elementary reactions are in near-equilibrium (this is called the quasi-steady-state assumption).

In contrast with an *in vitro* experiment, one major challenge with *in vivo* measurements of concentrations and reaction rates is the presence of significant error. Except for very low abundance metabolites, the errors are normally *relative*, meaning that they are proportional to the metabolite concentrations, instead of additive. (Relative error is shown in available experimental data in Additional file 1: Figure S1.) To quantify measurement precision with relative errors, experimentalists often use the coefficient of variation (CV), which is calculated by dividing the standard deviation of peak area/height by the mean peak area/height [4–6]. Methods such as least squares, which assume additive errors, are often not going to produce accurate estimates of parameters with relative errors. Because of such significant relative errors, it might not be reasonable to assume that errors are only in reaction rates as most of the *in vitro* enzyme kinetics methods assume. Relative errors in both concentrations and reaction rates need to be considered. Furthermore, many *in vivo* experiments are not time courses, so the data are assumed to be at steady-state. Another challenge with *in vivo* measurements is the difficulty of measuring enzyme and intermediate enzyme complex concentrations [7, 8], so these are typically unknown. Finally, control over metabolite concentrations in the cell is limited, so the range of experimental data points may be suboptimally distributed for accurate estimation of all parameters, making it difficult to estimate some parameters of a reaction.

A new estimation method, InVEst, standing for *In Vivo* Estimation, is described for estimating reaction parameters that addresses the specific challenges of *in vivo* data. InVEst uses maximum likelihood estimation, based on a model where all measurements have relative errors. As described, InVEst uses a family of reversible reaction mechanisms with multiple reactants and products with a single displacement mechanism. It is not always possible to obtain data from the entire range of metabolite concentrations and reaction rates, so some parameters may not be identifiable. InVEst estimates the standard deviations of parameter estimates using bootstrapping (a method of estimating variation in statistics by random subsampling of a data set), so that the user can understand the range of errors for the estimates.

Many methods for estimating kinetic parameters have been proposed, ranging from informal graphical plotting to sophisticated statistical non-linear regression methods. However, none have addressed all of the problems of *in vivo* estimation discussed above. Many methods are based on the Michaelis Menten equation which are normally applied to irreversible single substrate, single product reactions. Standard graphical plotting methods, such as the double reciprocal plot [9] and direct linear plot [10], are not based on statistical estimation and yield unnecessarily inaccurate parameter estimates. Some more statistically-based methods deal with relative error or errors in all measurements – but not both. Specifically, weighted least squares [11] is a general method often used in non-linear regression that can be applied to various kinds of reactions, however, it assumes the errors are additive and that only reaction rates have errors. Total least squares [12] improves ordinary least squares by dealing with errors in all measurements, but the errors are still assumed to be additive. Raaijmakers’ maximum likelihood estimation method [13] can deal with relative errors, but assumes that errors are in reaction rates only. Liebermeister et al. [14] have developed a method that integrates knowledge from many sources, along with *in vivo* measurements, to estimate kinetic parameters using Bayesian methods. However, this method still assumes only additive errors and requires a lot of prior information about the parameters. Only InVEst deals with relative errors in all measurements as well as reversible reactions with multiple substrates and products. A summary of existing methods appears in Table 1.

**Table 1:** Features of different enzyme kinetic parameter estimation methods. “WLS” stands for the weighted least squares method. “TLS” stands for the total least squares method. “Raaijmakers” is the maximum likelihood method of Raaijmakers

	Multiple substrates/	Reversible	Relative	Error in all
	products	reaction	error	variables
Double reciprocal	✗	✗	✗	✗
Direct linear	✗	✗	✓	✓
WLS	✓	✓	✗	✗
TLS	✓	✓	✗	✓
Raaijmakers	✗	✗	✓	✗
InVEst	✓	✓	✓	✓

In this paper, our goal is to focus on the specific problem of estimating kinetic parameters as accurately as possible, given realistic assumptions about data errors. We discuss the formulation of InVEst, and evaluate the method on simulated data. We show that InVEst works well on data with relative errors in all measurements. We also demonstrate the application of InVEst and discuss the parameter identifiability issue.

## Methods

Like most methods of kinetic parameter estimation, we assume that temperature and pressure are constant, so rate constants in mass action kinetic equations are constant, and the Gibbs Free Energy of Formation is constant. We also assume that the measured system is at steady state, meaning that the time derivatives of metabolite concentrations and reaction rates are zero.

Also, we assume that there are measurements of stable reactants and products of enzyme reactions, but not substrate-enzyme complexes, product-enzyme complexes and free enzyme concentrations, as they are generally difficult to measure experimentally. It is assumed that metabolite concentrations are obtained by high-throughput methods, such as chromatography, mass spectroscopy, or nuclear magnetic resonance spectroscopy [15]. For example, reasonably accurate concentration data can be obtained by mass spectroscopy with internal standards. Normally, average value of coefficient of variation for mass spectrometry below 0.2 is considered as good measurements [16–18], and thus it is not unreasonable to expect such data to have a constant coefficient of variation (i.e., normally distributed relative error) of 20 *%*.

We also assume that it is possible to obtain measurements of reaction rates. For steady state reaction rate measurement, one widely used method is *C*^13^ labeling, which uses a cell culture at steady state in a medium with labeled-carbon substrates. Reaction rates can be determined by analyzing the labeling pattern of targeted metabolites from mass spectrometry [19]. In addition, we assume that the Gibb’s Free Energies of Formation of metabolites are known, since these are used to compute the equilibrium constants (*K*_*eq*_) for enzymatic reactions.

### Single substrate and product reversible reactions

We use a standard simple but general reaction mechanism to represent most metabolic reversible reactions [20]. This subsection considers single reactant/product case. The more general case consisting of multiple reactants and multiple products will be discussed later. The reaction is a three step process, namely binding, conversion and release:

(1) $$ \mathrm{a} + \mathrm{E}\; {\underset{k_{\text{-}1}}{\overset{k_{1}}\rightleftharpoons}} \; \text{aE} \;{\underset{k_{\text{-}2}}{\overset{k_{2}}\rightleftharpoons}}\; \text{bE} \; {\underset{k_{\text{-}3}}{\overset{k_{3}}\rightleftharpoons}} \; \mathrm{b} + \mathrm{E}  $$

where *a* is the reactant, *b* is the product, *E* is the free enzyme, *aE* and *bE* are the intermediate complexes, and *k*_*i*_ and *k*_−*i*_ are reaction rate constants for *i*∈{1,2,3}.

Assuming the reaction is at steady state, an equation for the reaction rate can be written as:

(2) $$ v = \frac{K_{eq}[\!a]-[\!b]}{c_{1} + c_{2}[\!a] + c_{3}[\!b]}  $$

where $K_{\textit {eq}} = \frac {k_{1}k_{2}k_{3}}{k_{-1}k_{-2}k_{-3}}$ is an equilibrium constant, obtained from the Standard Gibbs Free Energy of Formation of the reactants and products.

*c*_1_ is

$$\left(\frac{k_{2}k_{3}}{k_{-1}k_{-2}k_{-3}} + \frac{k_{3}}{k_{-2}k_{-3}} + \frac{1}{k_{-3}}\right)/\left[E_{tot}\right], $$

*c*_2_ is

$$\left(\frac{k_{1}k_{2}}{k_{-1}k_{-2}k_{-3}} + \frac{k_{1}k_{3}}{k_{-1}k_{-2}k_{-3}} + \frac{k_{1}}{k_{-1}k_{-3}}\right)/[\!E_{tot}], $$

*c*_3_ is

$$\left(\frac{1}{k_{-2}} + \frac{1}{k_{-1}} + \frac{k_{2}}{k_{-1}k_{-2}}\right)/[\!E_{tot}], $$

and [ *E*_*tot*_], the total enzyme, is [ *E*]+[ *aE*]+[ *bE*].

If *K*_*eq*_ is very large and the reversible reactions’ rate constants (*k*_−2_ and *k*_−3_) are small, *c*_3_ can be neglected and the rate Eq. 2 can be reduced to standard irreversible Michaelis Menten equation.

This rate equation can be derived from the ordinary differential equations for mass action kinetics of a reaction (1), by setting the derivatives of the concentrations of all chemical species to zero (since the system is assumed to be at steady state) and solving for [ *E*_*tot*_]. The detailed derivation and calculation for the steady state equation and equilibrium constant are presented in Additional files 2 and 3.

### Parameter estimation by maximum likelihood for single substrate/product reversible reaction

The InVEst method estimates the parameters of kinetic rate Eq. (2) using maximum likelihood, assuming relative error in all measurements. Parameters are estimated from a set of *n* experiments, each with data values for *a*_*i*_ (substrate), *b*_*i*_ (product), *v*_*i*_ (reaction rate), for experiment *i*.

Each data value has some known relative error. Specifically, we have *a*_*i*_=*a*_*i*0_*ε*_*a*_, *b*_*i*_=*b*_*i*0_*ε*_*b*_ and *v*_*i*_=*v*_*i*0_*ε*_*v*_, where *a*_*i*0_, *b*_*i*0_, and *v*_*i*0_ are latent variables representing the data values without measurement error, multiplied by a normally distributed error with mean 1 and standard deviation *σ*: $\epsilon _{x} \sim N \left (1,{\sigma _{x}^{2}}\right)$ (where *x* is *a*, *b*, or *v*).

The likelihood function is:

$$\begin{aligned} L(a_{i0},b_{i0},v_{i0},c_{1},c_{2},c_{3} ; a_{i},b_{i},v_{i})= \\ f(a_{i},b_{i},v_{i} ; a_{i0},b_{i0},v_{i0},c_{1},c_{2},c_{3}) \end{aligned} $$

Since each data acquisition can be carried out independently [21], errors in *a*, *b* and *v* can be assumed to be independent of *c*_1_,*c*_2_ and *c*_3_ and each other, the likelihood function can be written as

$$\begin{aligned} & f(a_{i},b_{i},v_{i} ; a_{i0},b_{i0},v_{i0}) = \\ & \prod f(a_{i} ; a_{i0}) \prod f(b_{i} ; b_{i0}) \prod f(v_{i} ; v_{i0}) \end{aligned} $$

The distribution of *a*_*i*_ is

$$N\left(a_{i0},a_{i0}^{2}{\sigma_{a}^{2}}\right) = \frac{1}{\sqrt{2\pi a_{i0}^{2}{\sigma_{a}^{2}}}}\exp\left(-\frac{(a_{i}-a_{i0})^{2}}{2a_{i0}^{2}{\sigma_{a}^{2}}}\right) $$

The distributions of the other data values are similar.

The parameters that maximize the likelihood also maximize the log of the likelihood, which is

$${} {\fontsize{9.4pt}{9.6pt}\selectfont{\begin{aligned} \log(L)= &\sum_{i=1}^{n}\left(-\log\left(a_{i0}\sigma_{a}\sqrt{2\pi}\right)\right) + \sum_{i=1}^{n}\left(-\frac{(a_{i}-a_{i0})^{2}}{2a_{i0}^{2}{\sigma_{a}^{2}}}\right) \\ &+\sum_{i=1}^{n}\left(-\log\left(b_{i0}\sigma_{b}\sqrt{2\pi}\right)\right) + \sum_{i=1}^{n}\left(-\frac{(b_{i}-b_{i0})^{2}}{2b_{i0}^{2}{\sigma_{b}^{2}}}\right) \\ &+\sum_{i=1}^{n}\left(-\log\left(v_{i0}\sigma_{v}\sqrt{2\pi}\right)\right) +\sum_{i=1}^{n}\left(-\frac{(v_{i}-v_{i0})^{2}}{2v_{i0}^{2}{\sigma_{v}^{2}}}\right) \end{aligned}}} $$

Negating the log likelihood and dropping constant factors yields an objective function to minimize, subject to the constraints of Eq. 2.

$$\begin{aligned} \min &\left(\sum_{i=1}^{n}(\log(a_{i0})+\log(b_{i0})+\log(v_{i0})) \right.\\ &+ \frac{1}{2{\sigma_{a}^{2}}}\sum_{i=1}^{n}\left(\frac{a_{i}}{a_{i0}}-1\right)^{2} + \frac{1}{2{\sigma_{b}^{2}}}\sum_{i=1}^{n}\left(\frac{b_{i}}{b_{i0}}-1\right)^{2} \\ &\left. + \frac{1}{2{\sigma_{v}^{2}}}\sum_{i=1}^{n}\left(\frac{v_{i}}{v_{i0}}-1\right)^{2} \right)\\ \text{s.t.}& \quad v_{i0} = \frac{K_{eq}a_{i0} - b_{i0}}{c_{1} + c_{2}a_{i0} +c_{3}b_{i0}}, \text{\ where \(i = 1,2,\cdots,n\)} \end{aligned} $$

where all the *a*_*i*_, *b*_*i*_ and *v*_*i*_ are experimental measurements, all the relative errors *σ* are known and *a*_*i*0_, *b*_*i*0_, *v*_*i*0_ are latent variables, and *c*_1_, *c*_2_ and *c*_3_ are the parameters to be estimated by solving the optimization problem.

In the implementation, this is simplified to an unconstrained optimization problem by substituting the right-hand side of Eq. 2 for *v*_*i*0_.

### Generalization to multiple substrates and products

For reactions with multiple substrates and products, there are two possible mechanisms, namely single-displacement and double-displacement. For single-displacement reactions, the order of substrates binding to the enzyme can be random or ordered. Those two type of reactions can be approximated by following reaction [22]:

$$\begin{aligned} & \mathrm{a}_{1} + \mathrm{a}_{2} + \cdots + \mathrm{a}_{\mathrm{m}} + \mathrm{E} \; {\underset{k_{\text{-}1}}{\overset{k_{1}}\rightleftharpoons}} \; \mathrm{a}_{1}\mathrm{a}_{2}\cdots \mathrm{a}_{\mathrm{m}}\mathrm{E} \\ & {\underset{k_{\text{-}2}}{\overset{k_{2}}\rightleftharpoons}} \; \mathrm{b}_{1}\mathrm{b}_{2}\cdots \mathrm{b}_{\mathrm{p}}\mathrm{E}\; {\underset{k_{\text{-}3}}{\overset{k_{3}}\rightleftharpoons}} \;\mathrm{b}_{1} + \mathrm{b}_{2} + \cdots + \mathrm{b}_{\mathrm{p}} + \mathrm{E} \end{aligned} $$

where *m* is the number of reactants and *p* is the number of products in this reaction.

A steady state equation can be derived as in the single reactant/product case:

(3) $$ v = \frac{K_{eq}\prod\limits_{j=1}^{m}[\!a_{j}]-\prod\limits_{j=1}^{p}[\!b_{j}]} {c_{1} + c_{2}\prod\limits_{j=1}^{m}[\!a_{j}] + c_{3}\prod\limits_{j=1}^{p}[\!b_{j}]}  $$

where *c*_1_, *c*_2_, *c*_3_, *K*_*eq*_, and *E*_*tot*_ are as before.

The derivation of the objective function to minimize in order to find the parameters that maximize the likelihood is a straightforward generalization of the single substrate/product case.

$${} {\fontsize{9.4pt}{9.6pt}\selectfont{\begin{aligned} \min &\left(\sum_{i=1}^{n}\sum_{j=1}^{m}\log(a_{ij0}) +\sum_{i=1}^{n}\sum_{j=1}^{p}\log(b_{ij0}) +\sum_{i=1}^{n}\log(v_{i0}) \right.\\ &+ \frac{1}{2{\sigma_{a}^{2}}}\sum_{i=1}^{n}\sum_{j=1}^{m}\left(\frac{a_{ij}}{a_{ij0}}-1\right)^{2} + \frac{1}{2{\sigma_{b}^{2}}}\sum_{i=1}^{n}\sum_{j=1}^{p}\left(\frac{b_{ij}}{b_{ij0}}-1\right)^{2} \\ &+ \left. \frac{1}{2{\sigma_{v}^{2}}}\sum_{i=1}^{n}\left(\frac{v_{i}}{v_{i0}}-1\right)^{2} \right) \end{aligned}}} $$

which is maximized subject to the constraints of Eq. 3.

In the implementation, this can also be simplified to an unconstrained optimization problem by substituting the right-hand side of Eq. 3 for *v*_*i*0_.

### Parameter identifiability

It is sometimes not possible to obtain *in vivo* data whose values are well enough distributed to estimate all parameters accurately. In this section, we characterize some cases when parameters cannot be accurately estimated. From Eq. (2), it is clear that when one term in the denominator is much smaller than the others, *v* is relatively insensitive to the corresponding parameter. For example, if *c*_1_,*c*_2_*a*≫*c*_3_*b*, then Eq. 2 will be approximately

$$ v = \frac{K_{eq}[\!a]-[\!b]}{c_{1} + c_{2}[\!a]}, $$

So changes in *c*_3_ will have little effect on *v*. More importantly, changes in data values resulting from erroneous estimates of *c*_3_ will be small relative to the noise in the data, so estimates of *c*_3_ tend to have large errors. Similarly, estimates of *c*_1_ tend to have large errors when *c*_2_*a*+*c*_3_*b*≫*c*_1_ and estimates of *c*_2_ have large errors when *c*_1_+*c*_3_*b*≫*c*_2_*a*.

**Fig. 1:**
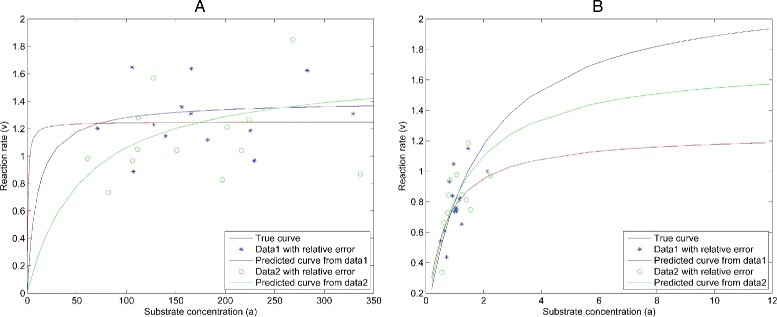
Identifiability issue in two parameter case. When data points are not well-distributed, parameter identification can be difficult. This shows the curve for parameters predicted from two possible data sets, one with points gathered in the saturation region (where reaction rates asymptotically approach a maximum level) in (**a**) and in the other in the linear region (where substrate concentrations are small and reaction rates increase almost linearly with substrate concentrations) in (**b**)

For illustration, consider the simpler case when *K*_*eq*_ is very large and the rate Eq. (2) can be approximated by the standard Michaelis Menten equation. In Fig. 1(a), two data sets derived from the same actual parameters have large *a*_*i*_, so the *v*_*i*_ values lie near the maximum value of the curve. We call this region as saturation region since reaction rates asymptotically approach a maximum level, and additional increases in the substrate concentration do not lead to an increase in the reaction rates. In this case, *c*_2_, which determines the maximum value, is the only parameter that affects the curve fit, so estimates of *c*_1_ from both data sets have large errors. In Fig. 1(b), all of the substrate concentration *a*_*i*_ values are small, so the points lie near the region where the curve is increasing linearly. We call this region as linear region since reaction rates increase in almost a linear fashion with increasing substrate concentrations. The slope in this region is determined by *c*_1_ almost independently of *c*_2_ so estimates of *c*_2_ have large errors.

Estimates of the accuracy of parameter estimates must be obtained using the available data. InVEst uses bootstrapping to estimate the variance of the parameter estimates.

### Bootstrap estimation of standard error

The *c* parameter estimates can vary widely in accuracy, depending on the experimental data. Bootstrapping [23] is used to estimate the relative standard errors and bias of the parameter estimates, so users can tell whether the parameter estimation is good or not. Let $\hat {c}$ be the estimate from the data, and $\hat {c_{i}}^{*}$ be the estimate from a bootstrap sample. A typical recommendation is to use *N*=*n*^2^ bootstrap samples for *n* experimental measurements [24]. The bootstrap estimation of standard errors is calculated from $SE_{B}(\hat {c}) = \left [\frac {1}{N}\sum (\hat {c_{i}}^{*}-\hat {c})^{2}\right ]^{\frac {1}{2}}$ and bias estimation is calculated by $\mathit {Bias} = \frac {1}{N}\sum \hat {c_{i}}^{*}-\hat {c}$[25]. As the *c* parameters have a large range of possible values, it is more appropriate to use relative errors and relative bias to describe the estimate. The relative standard error is calculated by $SE_{B}/\hat {c}$ and the relative bias is calculated by $\mathit {Bias}/\hat {c}$.

### Estimation of total enzyme change

Estimating kinetic parameters can be useful for identifying the effects of genetic changes or drug treatments that target metabolic enzymes. The total concentration of the enzyme in the cell may change because of changes in gene expression or loss of function in one or more copies of the gene coding for the enzyme, or the activity may change because of changes in the protein sequence or post translational modifications. Estimating these changes for specific enzymes in each sample can help identify the target of a mutation or drug (it’s the enzyme whose activity changes the most), and may be useful for estimating the impact of such a change on flux through a network.

Since each of the kinetic parameters *c*_*i*_ is of the form *ci*′/*E*_*tot*_, where *ci*′ is independent of the enzyme concentration, *E*_*tot*_ can be estimated from the ratio

$$ \frac{E_{tot}^{wt}}{E_{tot}^{mt}} = \frac{c_{i}^{mt}}{c_{i}^{wt}} $$

where $c_{i}^{wt}$ and $c_{i}^{mt}$ are corresponding *c*_*i*_ parameters (*i* = 1, 2 or 3) for wild type and mutant (or drug treated) samples. Note that it is possible to obtain a reliable estimate for *E*_*tot*_ whenever there are reliable estimates for one of the three parameters in both samples.

## Results

### Evaluate InVEst using simulated data

We evaluate the parameter estimation method on simulated data. For MATLAB code for reproducing the results of this work, please refer to [26]. The simulations were carried out in MATLAB on a laptop computer with an Intel Core i5-4200u 2.3 GHz processor and 8 GB installed memory.

Many reactions in metabolic pathways have multiple substrates and products and are reversible reactions. The simulation is based on the reaction acetylornithine aminotransferase from *Saccharomyces Cerevisiae* Arginine biosynthesis pathway with Arg8 [27]. Kinetic parameters and the total enzyme concentration are not available, and thus we use some heuristic numbers for them. The experimental data are chosen to be well-distributed, since poorly distributed data would guarantee inaccurate parameter estimates even for the best possible estimation method.

The reaction is:

$$\text{AcGLU-SA} + \text{GLU} \rightleftharpoons \text{AcORN} + {2-\text{oxoglutarate}} $$

Abbreviations [28]: AcGLU-SA, N-acetyl-glutamate-semialdehyde; GLU, L-glutamate; AcORN, N-acetyl- ornithine.

The standard Gibbs Free Energy of Formation for the metabolites are taken from MetaCyc database [29], and are provided in the Additional file 4. The standard Gibbs Free Energy of Formation can be used to compute *K*_*eq*_=1.7281, and, assuming *E*_*tot*_=1 *M*, the *c* parameters are *c*_1_=2.5783, *c*_2_=3.7327 and *c*_3_=3.5238.

To characterize the amount of data for effective use of InVEst, we evaluated the accuracy of parameter estimates for varying numbers of simulated experiments. Data sets of 12, 24 and 30 experiments were generated by choosing values for substrate and product concentrations and computing *v* exactly for each choice based on Eq. 3. Relative errors were introduced by multiplying a random value from the normal distribution of *N*(1,*σ*^2^). A value of 0.2 was used for *σ* for metabolites, and *σ*_*v*_ of 0.2 was used for reaction rates.

For each number of experiments, 1,000 simulated data sets were generated, the *c* parameters were estimated using InVEst, and the mean and standard error were calculated. The results are shown in Table 2. With increasing sample size, the relative standard errors and bias in the estimates are improved. It is evident that the results for sample size of 24 and 30 are quite accurate with relative standard error near 10 % and very small relative bias. Twenty to thirty samples seems to be a reasonable sample size to choose for accurate estimations.

**Table 2:** Average *c* parameter estimates, relative standard errors and relative bias as a function of number of experiments for acetylornithine aminotransferase when *σ*_*v*_=0.2. Results are based on 1,000 simulated data sets. “n” is the number of experiments. “Avg Est” is the average value of the estimates. “Rel SE” is the relative standard error, and “Rel bias” is the relative bias

n	Run time	True	Avg Est	Rel SE	Rel bias
12	1.74sec/simulation	*c*_1_:2.578	2.315	0.188	0.102
		*c*_2_:3.733	3.68	0.108	0.014
		*c*_3_:3.524	3.54	0.10	0.007
24	7.98sec/simulation	*c*_1_:2.578	2.567	0.143	0.004
		*c*_2_:3.733	3.755	0.081	0.006
		*c*_3_:3.524	3.544	0.087	0.006
30	20.04sec/simulation	*c*_1_:2.578	2.573	0.129	0.002
		*c*_2_:3.733	3.742	0.062	0.002
		*c*_3_:3.524	3.517	0.073	0.002

Second, we consider the effect of greater error in reaction rate estimates, with *σ*_*v*_=0.5. The results are shown in Table 3. The relative standard errors increase, but are still below 20 %. The relative bias values are also low. This shows that InVEst is robust to different measurement errors.

**Table 3:** *c* parameter estimates for acetylornithine aminotransferase when *σ*_*v*_=0.5. Results are based on 1000 simulated data sets of 30 experiments, each

	True	Avg Est	Rel SE	Rel bias
*c*_1_	2.578	2.555	0.189	0.009
*c*_2_	3.733	3.806	0.127	0.020
*c*_3_	3.524	3.652	0.140	0.036

It is also possible to evaluate the accuracy of estimates when there is only one data set (with multiple experiments) available, as would be the case in normal use of InVEst in practice. The bootstrap method is used to estimate relative standard errors in parameter estimates. To evaluate the bootstrap method, we generated a single data set of 30 experiments as the input data for parameter estimation and randomly subsampled the 30 data points 1,000 times. Each bootstrap subsample simulation took around 10 sec. The estimates for *σ*_*v*_=0.2 and *σ*_*v*_=0.5 are shown in Tables 4 and 5 respectively. As expected, the bootstrap estimates are very similar to the previous estimates from 1,000 simulated data sets.

**Table 4:** *c* parameter estimates for acetylornithine aminotransferase when *σ*_*v*_=0.2. Estimates are from a single simulated data set of 30 experiments. The bootstrap method was used to estimate relative standard error (“Rel SE”) and relative bias (“Rel bias”)

	True	Est	Rel SE	Rel bias
*c*_1_	2.578	2.750	0.111	0.008
*c*_2_	3.733	3.902	0.066	0.005
*c*_3_	3.524	3.552	0.094	0.016

**Table 5:** *c* parameter estimates for acetylornithine aminotransferase when *σ*_*v*_=0.5. Estimates are from a single simulated data set of 30 experiments. The bootstrap method was used to estimate relative standard error (“Rel SE”) and relative bias (“Rel bias”)

	True	Est	Rel SE	Rel bias
*c*_1_	2.578	2.933	0.152	0.021
*c*_2_	3.733	4.081	0.184	0.052
*c*_3_	3.524	3.343	0.243	0.059

### Comparison of InVEst with prior methods

Most current methods produce optimal estimates only when errors are additive and when errors occur only in reaction rate measurements. These assumptions are generally not true with *in vivo* data. In this subsection, we compare InVEst to some existing methods and show that InVEst produces better estimates when data have relative errors in all measurements.

As some of the existing methods only work on irreversible enzymatic reactions, we use the two parameter case of Eq. 2 for comparison. In this case, there are two parameters to be estimated, namely *c*_1_ and *c*_2_.

$$v = \frac{K_{eq}[\!a]}{c_{1} + c_{2}[\!a]}, $$

We first simulate the data with relative errors to both substrate *a* and reaction rate *v*, and second apply InVEst and prior methods to obtain estimates for the Michaelis Menten like curve. One thousand simulated data sets of 30 experiments each are used. The results are summarized in Table 6. InVEst has superior performance in the estimates and relative standard errors.

**Table 6:** Comparison of the accuracy of prior methods: total least square (TLS), ordinary least square (OLS), direct linear plot (DLP), double reciprocal plot(DRP) and InVEst. True *c*_1_=1.5, True *c*_2_=0.8. Data have relative errors in all variables. Results are based on 1,000 simulated data sets of 30 experiments, each. “Avg Est” is the average value of the estimates. “Rel SE” is the relative standard error

	Avg Est *c*_1_	Avg Est *c*_2_	Rel SE *c*_1_	Rel SE *c*_2_
TLS	0.840	0.940	0.389	0.143
OLS	1.036	0.921	0.413	0.147
DLP	1.396	0.883	0.429	0.262
DRP	1.859	0.498	0.307	1.124
InVEst	1.518	0.766	0.128	0.112

### Predicting total enzyme concentration change

As noted above, the relative difference in *E*_*tot*_ between wild type and mutant or drug-treated samples can be estimated from the estimate of any of the *c*_*i*_ parameters from two sets of experiments.

$$\frac{E_{tot}^{wt}}{E_{tot}^{mt}} = \frac{c_{i}^{mt}}{c_{i}^{wt}}. $$

We illustrate estimation of *E*_*tot*_ change using the Arg8 reaction. For the wild type samples, the total enzyme concentration is $E_{\textit {tot}}^{wt} = 1 \hspace {1 mm} M$, and for the mutant/drug treated samples, the total enzyme concentration is $E_{\textit {tot}}^{mt} = 0.1\hspace {1mm} M$. Results of the wild-type estimate appear in the previous section. Additional data for the mutant were generated as above based on the *c* parameter values of mutant/drug treated sample and 1,000 simulated data sets are used. The estimates for mutant/drug treated sample are shown in Table 7.

**Table 7:** *c* parameter estimates for acetylornithine aminotransferase from mutant/drug treated sample. Results are based on 1,000 simulated data sets

	True	Avg Est	Rel SE	Rel bias
*c*_1_	25.783	24.784	0.119	0.039
*c*_2_	37.327	37.480	0.061	0.004
*c*_3_	35.238	35.518	0.065	0.008

To obtain the prediction of total enzyme change, we take $c_{i}^{mt}/c_{i}^{wt}$. The results are shown in Table 8.

**Table 8:** *E*_*tot*_ change prediction based on 1,000 simulated data sets

	True	Avg Est	Rel SE	Rel bias
$\frac {E_{\textit {tot}}^{wt}}{E_{\textit {tot}}^{mt}} = \frac {c_{1}^{mt}}{c_{1}^{wt}}$	10	10.214	0.091	0.021
$\frac {E_{\textit {tot}}^{wt}}{E_{\textit {tot}}^{mt}} = \frac {c_{2}^{mt}}{c_{2}^{wt}}$	10	9.957	0.022	0.004
$\frac {E_{\textit {tot}}^{wt}}{E_{\textit {tot}}^{mt}} = \frac {c_{3}^{mt}}{c_{3}^{wt}}$	10	10.115	0.049	0.012

Since any of the *c*_*i*_ parameters can be used to estimate the change in *E*_*tot*_, the one that gives minimum standard error, *c*_2_, was chosen. This also demonstrates that even though sometimes identifiability issues can occur and some parameters cannot be estimated, our method could still be very useful if one parameter can be estimated accurately.

## Discussion

This work is intended to be a first step towards estimating parameters for reactions in large metabolic networks *in vivo*. *In vivo* estimation will need to be based on data that have relatively large relative errors in all measured parameters, and will have to deal with a variety of reaction kinetics, including reactions that are reversible and have multiple substrates and/or products. Although measurement and estimation of enzyme kinetics has been studied for many decades, there is no single existing estimation method that addresses all of these issues. We have proposed a maximum likelihood approach to estimate kinetic parameters using nonlinear optimization, with estimates on the standard error and bias of the results using the bootstrap.

Simulations show that InVEst produces accurate estimates for realistic high-throughput metabolomics data. For example, with 20–30 samples with coefficients of 20 % in metabolite concentrations and 50 % in reaction rate estimates, estimates have a relative standard error of less than 20 %. Collecting data of this quality would be technically difficult, but is within the current state of the art.

An advantage of the method is that it estimates each set of reaction parameters independently. If measurements are not available for some metabolites, it can still estimate parameters for those reactions for which the data include all substrates and products.

Solving the problem of *in vivo* parameter estimation in its full generality will require meeting a number of additional challenges. Some reactions have more complex kinetics than those we consider, especially various kinds of inhibition. When the inhibiting metabolite and mechanism of inhibition are known, the approach described here can probably be generalized to accommodate the inhibition mechanism in our future work. Otherwise, a process of *model selection* may be necessary, where competing models are estimated and the quality of the results compared, with appropriate adjustments for model complexity. In addition, it will be necessary to deal with the kinetics of transport reactions, and to take account of different compartments in the cell.

Parameter identifiability is a difficult issue in *in vivo* estimation. We have shown that accurate estimates of all parameters require data that is well-distributed over the kinetics curve, but such data will not often be obtainable for several reasons. Experimental data must be obtained by perturbing metabolites and fluxes, for example, by adjusting nutrient media, testing mutants, and targeting reactions with drugs. First, accurate estimation may require non-physiological concentrations of metabolites – estimating *c*_3_ for a reaction that is nearly irreversible being an example. More generally, there is usually inadequate controllability of metabolite concentrations and reaction fluxes to obtain the experimental values needed for accurate estimation, for many reasons including concentrations are toxic or inadequate to sustain life, and rate-limiting reactions that make high fluxes in other reactions impossible to obtain. Since we can’t estimate everything accurately, it is important to produce estimates of the standard errors of parameter estimates, so we can tell which ones are meaningful. Also, as we note above, if some but not all parameters of a reaction can be estimated accurately, the results still may be useful. For example, it is possible to estimate the total concentration or relative activity of an enzyme in wild-type vs. mutant cells when only one of the kinetic parameters is accurately estimated.

## Conclusion

In conclusion, a new method, InVEst, is developed for estimating reaction kinetic parameters in metabolic networks that addresses the specific challenges of *in vivo* data. InVEst uses maximum likelihood estimation based on models where all measurements have potentially relative errors. It can be applied to multiple substrate/product reversible enzymatic reactions with a generalized single displacement mechanism. Because it is not always possible to obtain good data covering full range of possible metabolite concentrations and reaction rates, certain parameters may be non-identifiable. InVEst uses bootstrap to estimate the standard errors of parameter estimations that can tell which estimates are reliable.

InVEst enables the estimation of reaction rates directly from concentration measurements. Also, comparing the estimated kinetic parameters of a reaction in the wild type and mutant cells can quantify enzyme abundance or activity change due to genetic variants. The same method can also be used to measure the effect of a drug that targets a particular enzyme. Moreover, estimated parameters can be used to estimate maximum reaction rates, which could be used as constraints to improve flux-balance analysis.

## Additional files

Additional file 1
**Experimental data support on relative error model.** **Figure S1** Noise errors in high-throughput metabolomic data tend to be relative. The plot shows the empirical standard deviation vs. mean of metabolite concentrations in a publicly available mass spectrometry data set of 40 human urine samples [30]. Each sample has 3 technical replicates, which were used to calculate the standard deviation and mean of metabolite concentrations. The data for “peak 105” were chosen because the chromatographic peak appears in all three replicates of the sample and the measurements cover a wide range of concentrations across different samples. Low concentrations are omitted because they are highly inaccurate due to background noise. There is a linear relationship (*R* ^2^=0.71) between standard deviation and concentration mean, showing that errors are proportional to measured concentration. (PDF 1894 kb)

Additional file 2
**Derivation for steady state rate equation.** This file provides a detailed derivation for steady state rate equation of a single reactant and single product reversible metabolic reaction presented in Methods section. (PDF 92.8 kb)

Additional file 3
**Equilibrium constant** ***K***_***eq***_ **.** The equilibrium constant *K*_*eq*_ is assumed to be a known constant. This file provides the calculation of equilibrium constant *K*_*eq*_ based on standard Gibbs Free Energies of Formation. (PDF 98.8 kb)

Additional file 4
**Standard Gibbs Free Energy of Formation MetaCyc.** This file provides standard Gibbs Free Energy of Formation taken from MetaCyc database [29] for metabolites used in the simulation example in Results section. (PDF 51.4 kb)
