# Supplementary figures and images for: Towards in vivo estimation of reaction kinetics using high-throughput metabolomics data: a maximum likelihood approach
Source: BMC Syst Biol. 2015 Oct 5;9:66. doi: 10.1186/s12918-015-0214-7 (PMC4595320; doi:10.1186/s12918-015-0214-7)

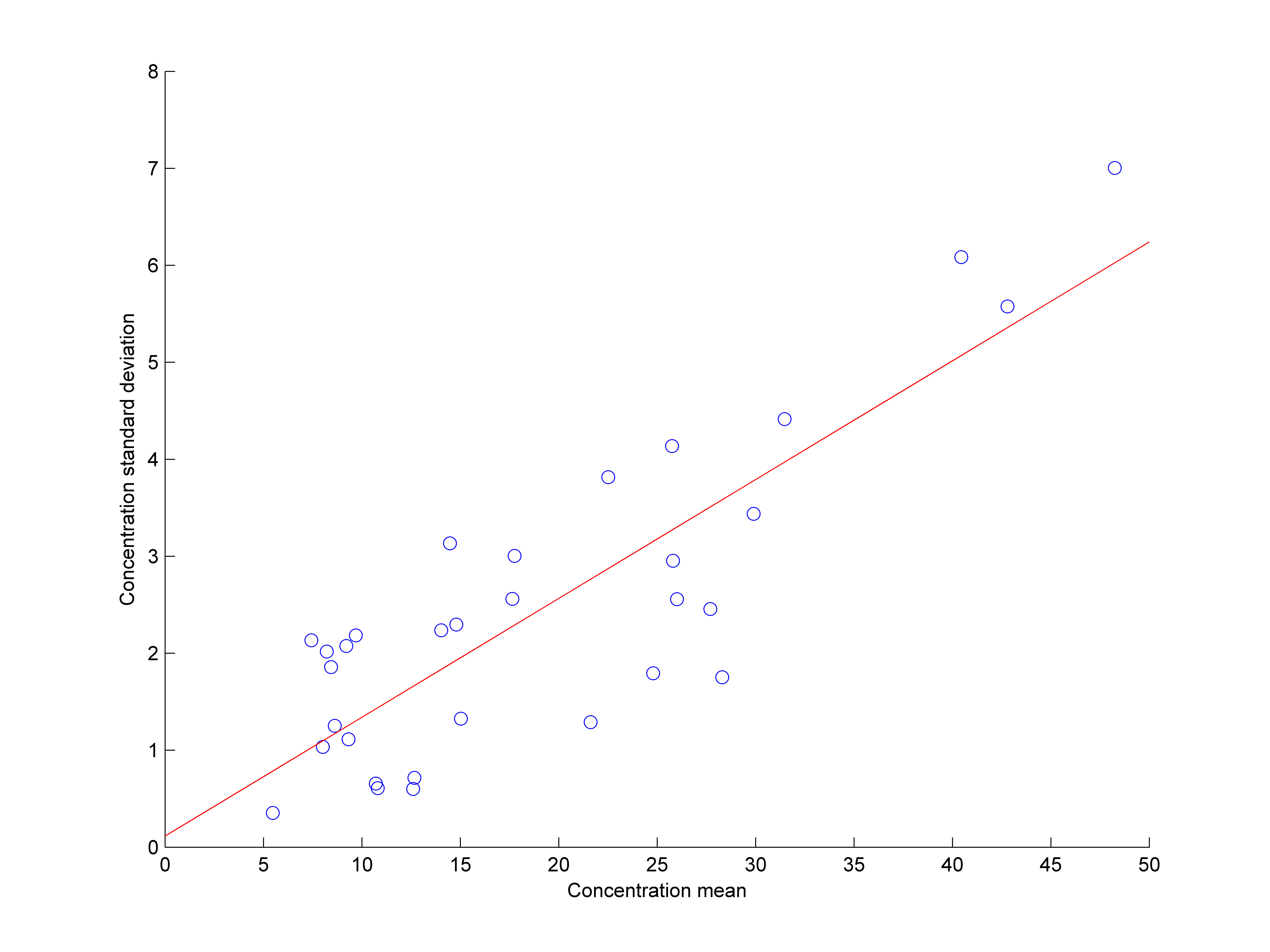

Supplement: Additional file 1 — Experimental data support on relative error model. Figure S1 Noise errors in high-throughput metabolomic data tend to be relative. The plot shows the empirical standard deviation vs. mean of metabolite concentrations in a publicly available mass spectrometry data set of 40 human urine samples [30]. Each sample has 3 technical replicates, which were used to calculate the standard deviation and mean of metabolite concentrations. The data for “peak 105” were chosen because the chromatographic peak appears in all three replicates of the sample and the measurements cover a wide range of concentrations across different samples. Low concentrations are omitted because they are highly inaccurate due to background noise. There is a linear relationship (R 2=0.71) between standard deviation and concentration mean, showing that errors are proportional to measured concentration. (PDF 1894 kb) [file 12918_2015_214_MOESM1_ESM.jpg]
