# Supplementary material for: Towards in vivo estimation of reaction kinetics using high-throughput metabolomics data: a maximum likelihood approach
Source: BMC Syst Biol. 2015 Oct 5;9:66. doi: 10.1186/s12918-015-0214-7 (PMC4595320; doi:10.1186/s12918-015-0214-7)
Supplement: Additional file 2 — Derivation for steady state rate equation. This file provides a detailed derivation for steady state rate equation of a single reactant and single product reversible metabolic reaction presented in Methods section. (PDF 92.8 kb) [file 12918_2015_214_MOESM2_ESM.pdf]

Additional file 2 — Derivation for steady state rate equation

Our reaction:

$$\mathrm{a} + \mathrm{E} \underset{k_{-1}}{\overset{k_1}{\rightleftharpoons}} \mathrm{aE} \underset{k_{-2}}{\overset{k_2}{\rightleftharpoons}} \mathrm{bE} \underset{k_{-3}}{\overset{k_3}{\rightleftharpoons}} \mathrm{b} + \mathrm{E}$$

This reaction has reactant a, and product b. E is the free enzyme, and aE and bE are the intermediate complexes. The three steps have k_1, $k_{\text{-}1}$, k_2, $k_{\text{-}2}$, k_3, and $k_{\text{-}3}$ as the reaction rate constants respectively. We also have the total enzyme concentration E_{tot} which is unchanged. InVEst requires the reactant concentrations, product concentrations and reaction rate v as inputs. These differential equations follow from the Law of Mass Action:

$$\begin{aligned}
\frac{d[a]}{dt} &= v_{in} - k_1[a][E] + k_{\text{-}1}[aE] \\
\frac{d[b]}{dt} &= -k_{\text{-}3}[b][E] + k_3[bE] - v_{out} \\
\frac{d[aE]}{dt} &= k_1[a][E] - k_{\text{-}1}[aE] - k_2[aE] + k_{\text{-}2}[bE] \\
\frac{d[bE]}{dt} &= k_2[aE] - k_{\text{-}2}[bE] - k_3[bE] + k_{\text{-}3}[b][E] \\
\frac{d[E]}{dt} &= -k_1[a][E] + k_{\text{-}1}[aE] - k_{\text{-}3}[b][E] + k_3[bE]
\end{aligned}$$

where v_{in} and v_{out} represent the incoming flux and outgoing flux respectively.

$[a]$, $[b]$, $[aE]$, $[bE]$ and $[E]$ are the concentrations. Total enzyme is divided between free enzyme $[E]$ and enzyme that is bound in intermediate complexes $[aE]$ and $[bE]$. The total enzyme concentration E_{tot} is assumed to be unchanged.

$$[E] + [aE] + [bE] = [E_{tot}]$$

To obtain the steady state solutions, differential equations are equated to 0. v_{in} and v_{out} are set to the steady state reaction rate v.

$$\begin{aligned}
v &= k_1[a][E] - k_{\text{-}1}[aE] \\
v &= k_2[aE] - k_{\text{-}2}[bE] \\
v &= k_3[bE] - k_{\text{-}3}[b][E] \\
[E_{tot}] &= [E] + [aE] + [bE]
\end{aligned}$$

Because we assume that we do not have the data for $[E]$, $[aE]$ and $[bE]$,

we solve the above polynomials to obtain expressions for them.

$$[E] = \frac{(k_2k_3 + k_{-1}k_3 + k_{-1}k_{-2})v}{k_1k_2k_3[a] - k_{-1}k_{-2}k_{-3}[b]}$$
$$[aE] = \frac{((k_1k_3 + k_1k_{-2})[a] + k_{-2}k_{-3}[b])v}{k_1k_2k_3[a] - k_{-1}k_{-2}k_{-3}[b]}$$
$$[bE] = \frac{((k_{-1}k_{-3} + k_2k_{-3})[b] + k_1k_2[a])v}{k_1k_2k_3[a] - k_{-1}k_{-2}k_{-3}[b]}$$

Given $[E_{tot}] = [E] + [aE] + [bE]$, we can add up the three concentrations of $[E]$, $[aE]$ and $[bE]$ to obtain the following equation:

$$[E_{tot}] = \frac{(k_2k_3+k_{-1}k_3+k_{-1}k_{-2})v+(k_1k_3+k_1k_{-2}+k_1k_2)[a]v+(k_{-1}k_{-3}+k_2k_{-3}+k_{-2}k_{-3})[b]v}{k_1k_2k_3[a]-k_{-1}k_{-2}k_{-3}[b]}$$

As the equilibrium constant $K_{eq} = \frac{k_1k_2k_3}{k_{-1}k_{-2}k_{-3}}$ is a known constant, we can simplify the equation and obtain:

$$v = \frac{K_{eq}[a]-[b]}{c_1+c_2[a]+c_3[b]}$$

where

$$c_1 = (\frac{k_2k_3}{k_{-1}k_{-2}k_{-3}} + \frac{k_3}{k_{-2}k_{-3}} + \frac{1}{k_{-3}})/[E_{tot}]$$
$$c_2 = (\frac{k_1k_2}{k_{-1}k_{-2}k_{-3}} + \frac{k_1k_3}{k_{-1}k_{-2}k_{-3}} + \frac{k_1}{k_{-1}k_{-3}})/[E_{tot}]$$
$$c_3 = (\frac{1}{k_{-2}} + \frac{1}{k_{-1}} + \frac{k_2}{k_{-1}k_{-2}})/[E_{tot}]$$
