# Supplementary material for: Towards in vivo estimation of reaction kinetics using high-throughput metabolomics data: a maximum likelihood approach
Source: BMC Syst Biol. 2015 Oct 5;9:66. doi: 10.1186/s12918-015-0214-7 (PMC4595320; doi:10.1186/s12918-015-0214-7)
Supplement: Additional file 3 — Equilibrium constant K eq . The equilibrium constant K eq is assumed to be a known constant. This file provides the calculation of equilibrium constant K eq based on standard Gibbs Free Energies of Formation. (PDF 98.8 kb) [file 12918_2015_214_MOESM3_ESM.pdf]

Additional file 3 — Equilibrium constant K_{eq}

At equilibrium, the Gibbs Free Energy:

$$\Delta G = 0 = \Delta G^0_{r1} + RT \ln \frac{[aE]}{[a][E]}$$
$$\Delta G = 0 = \Delta G^0_{r2} + RT \ln \frac{[bE]}{[aE]}$$
$$\Delta G = 0 = \Delta G^0_{r3} + RT \ln \frac{[b][E]}{[bE]}$$

where ΔG^0_r's are the standard Gibbs Free Energy of reactions. ΔG^0_{r1} is for the first step of binding. ΔG^0_{r2} is for the second step of conversion. ΔG^0_{r3} is for the third step of releasing.

Furthermore, we have the following equilibrium relations:

$$\frac{k_1}{k_{\text{-}1}} = \frac{[aE]}{[a][E]}$$

$$\frac{k_2}{k_{\text{-}2}} = \frac{[bE]}{[aE]}$$

and

$$\frac{k_3}{k_{\text{-}3}} = \frac{[b][E]}{[bE]}$$

Also because:

$$\Delta G^0_{r1} = \Delta G^0_{f[aE]} - (\Delta G^0_{f[a]} + \Delta G^0_{f[E]})$$
$$\Delta G^0_{r2} = \Delta G^0_{f[bE]} - \Delta G^0_{f[aE]}$$
$$\Delta G^0_{r3} = \Delta G^0_{f[b]} + \Delta G^b_{f[E]} - \Delta G^0_{f[bE]}$$

Where ΔG^0_f's are the standard Gibbs Free Energy of Formation which can be found out from databases such as MetaCyc. The Gibbs Free Energy change of the three steps is:

$$\Delta G^0_{r1} + \Delta G^0_{r2} + \Delta G^0_{r3} = -RT \ln(\tfrac{k_1}{k_{-1}} \cdot \tfrac{k_2}{k_{-2}} \cdot \tfrac{k_3}{k_{-3}}) = \Delta G^0_{f[b]} - \Delta G^0_{f[a]}$$

Thus, we can obtain $K_{eq} = \frac{k_1}{k_{-1}} \cdot \frac{k_2}{k_{-2}} \cdot \frac{k_3}{k_{-3}} = \exp(-\frac{\Delta G^0_{f[b]} - \Delta G^0_{f[a]}}{RT})$
