# Supplementary material for: Towards in vivo estimation of reaction kinetics using high-throughput metabolomics data: a maximum likelihood approach
Source: BMC Syst Biol. 2015 Oct 5;9:66. doi: 10.1186/s12918-015-0214-7 (PMC4595320; doi:10.1186/s12918-015-0214-7)
Supplement: Additional file 4 — Standard Gibbs Free Energy of Formation MetaCyc. This file provides standard Gibbs Free Energy of Formation taken from MetaCyc database [29] for metabolites used in the simulation example in Results section. (PDF 51.4 kb) [file 12918_2015_214_MOESM4_ESM.pdf]

Additional file 4 — Standard Gibbs Free Energy of Formation of Metabolite in Acetylornithine Aminotransferase

Table S4.1: Standard Gibbs Free Energy of Formation MetaCyc

Name	ΔG_f^0 kcal/mol
N-acetyl-glutamate-semialdehyde	-38.63
Glutamate	-82.27
N-acetylornithine	28.54
2-oxoglutarate	-149.76
